# Evaluation of the Chagas Western Blot IgG Assay for the Diagnosis of Chagas Disease

**DOI:** 10.3390/pathogens10111455

**Published:** 2021-11-10

**Authors:** Jean-Yves Brossas, Ballering Griselda, Margarita Bisio, Jeremy Guihenneuc, Julián Ernesto Nicolás Gulin, Stéphane Jauréguiberry, François-Xavier Lescure, Arnaud Fekkar, Dominique Mazier, Jaime Altcheh, Luc Paris

**Affiliations:** 1 Service de Parasitologie-Mycologie, APHP Sorbonne Université Hôpital Pitié-Salpêtrière, 75013 Paris, France; arnaud.fekkar@aphp.fr (A.F.); luc.paris@aphp.fr (L.P.); 2 Instituto Multidisciplinario de Investigación en Patologías Pediátricas (IMIPP), CONICET-GCBA, Buenos Aires C1425EFD, Argentina; g.ballering@gmail.com; 3 Servicio de Parasitología y Enfermedad de Chagas, Hospital de Niños “Dr. Ricardo Gutiérrez”, Instituto Multidisciplinario de Investigación en Patologías Pediátricas (IMIPP), CONICET-GCBA, Buenos Aires C1425EFD, Argentina; marguib@gmail.com (M.B.); ernestogulin@hotmail.com.ar (J.E.N.G.); jaltcheh@gmail.com (J.A.); 4 UMRS 1135, Centre d’Immunologie et des Maladies Infectieuses, Sorbonne Université, 75013 Paris, France; jeremy.guihenneuc@u-psud.fr (J.G.); dominique.mazier@upmc.fr (D.M.); 5 Service des Maladies Infectieuses et Tropicales, APHP, Sorbonne Université Hôpital Pitié-Salpêtrière, 75013 Paris, France; Stephane.jaureguiberry@aphp.fr; 6 Service des Maladies Infectieuses et Tropicales, APHP, Hôpitaux Universitaires Paris Nord val de Seine, Hôpital Bichat, 75000 Paris, France; francois-xavier.lescure@aphp.fr

**Keywords:** chagas disease, diagnostic, *Trypanosoma cruzi*

## Abstract

Chagas disease is a debilitating and often fatal pathology resulting from infection by the protozoan parasite *Trypanosoma cruzi*. In its recommendations, the World Health Organization states that the diagnosis of *T. cruzi* infection is usually based on the detection of antibodies against *T. cruzi* antigens and performed with two methodologically different assays. An inconclusive result can be resolved with a third “confirmatory” assay. The objective of this article is to evaluate the effectiveness of the Chagas Western Blot IgG assay (LDBio Diagnostics, Lyon, France) as a confirmatory serologic test. The Chagas Western Blot IgG assay was performed with native antigens derived from a *T. cruzi* strain of the TcVI genotype. Retrospective sera were provided by two parasitology laboratories (France and Argentina). The sensitivity, specificity, positive predictive value and negative predictive value of the Chagas blot were all 100% in our sera collection. The Chagas blot is an easy and qualitative method for the diagnosis of Chagas disease, with results in less than 2 h. This immunoblot has potential as a supplemental test for the confirmation of the presence of antibodies against *T. cruzi* in serum specimens. Nonetheless, the very good initial results presented here will need to be confirmed in larger studies.

## 1. Introduction

Chagas disease (American trypanosomiasis) is a parasitic infection endemic to the South American continent, where it affects millions of people and is responsible for thousands of deaths every year. *T. cruzi* vectors belong to Triatominae, which contains more than 140 species. Although most triatomine species are distributed in intertropical areas, the impact of climate change on the geographical distribution of vectors of Chagas disease could be important. Recent studies showed that certain species of triatomine have adapted and now also colonize different temperate regions in America. Before this adaptation of these vectors of *T. cruzi*, these regions were free from Chagas disease cases [1,2].

In the same way, worldwide migration and the displacement of populations lead infected but asymptomatic people to travel or settle in countries where the disease is not active. 

In many countries outside the endemic area, general practioners do not know the disease; this situation may be responsible for a significant delay in diagnosis when the chronic phase occurs.

Whereas the acute phase can be easily diagnosed with a direct blood exam (thick drop smear) or molecular technics, the chronic phase is essentially diagnosed by indirect methods, because *T. cruzi* is not abundant in the blood at this stage. More precisely, these indirect methods are based on the detection of antibodies but hampered by the absence of a gold standard method and the existence of false-positive results. The latter are notably described in the course of other parasitic diseases, such as cross-reactivity with Leishmania spp. A diagnosis reference method requires being easily accessible (therefore commercialized), sensitive, specific and applicable without major technical constraints. In this perspective, an immunoblot-based approach, the Chagas Western Blot IgG Assay^®^, which could meet these requirements, was developed by LDBio Diagnostics, Lyon, France. In the present work, we aim to evaluate this method within the setting of an international bicentric study.

## 2. Results

This case–control analytical study considered 278 patient sera provided by the Laboratory of Parasitology and Mycology of Pitié Salpêtrière Hospital (Paris, France) and the Laboratory of Parasitology and Chagas Disease of the Ricardo Gutiérrez Children’s Hospital (Buenos Aires, Argentina).

### 2.1. Patients’ Characteristics

Given that there is no gold standard for the diagnosis of Chagas disease, and in order to determine the diagnostic value of the Chagas Western Blot IgG assay for *T. cruzi* antibody detection, cases were selected by combining the biological characteristics (serological assays) and anamnestic data for each patient. The clinical and demographic characteristics of the patients were divided into two groups (Table 1). The positive group (Group 1) comprised 100 patients with a documented history of Chagas disease, active or not: 24 males and 73 females, with a median age of 34 years, and three undetermined gender (positive controls from an external quality control program). The youngest was a newborn aged one day and the oldest a male patient aged 78 years. All Group 1 patients originated from endemic areas. There were also sera from three babies born in France from a mother with a history of documented Chagas disease. For Group 1, the serologic expected result was positive. The control group (Group 2) was made up of 80 healthy subjects free of any parasitic infections (including 20 blood donors from French Polynesia) and 98 patients who were free of anti-*T. cruzi* antibodies but affected by other parasitic infections, i.e., acute toxoplasmosis (*n* = 28), leishmaniasis (*n* = 44), visceral amebiasis (*n* = 7) and malaria (*n* = 19). Group 2 had 78 male and 55 female sera but also 45 sera with no available gender information (principally leishmaniosis patients). The median age in Group 2 was 37 years, with the youngest patient a 10-month-old infant and the oldest a 77-year-old male.

### 2.2. Performance of the Chagas Western Blot IgG Assay^®^ in Comparaison with Two Other Commercial Assays

Appendix A details information about the Chagas Western blot IgG assay and data analysis. As described in Appendix A, the immunoblots were considered positive when at least two of the six bands of interest were present. 

For the Chagas Western Blot IgG assay, as indicated in Table 2, the sera from all Group 1 patients were positive. The immunoblot patterns for the control group (Group 2) showed no reactivity with any of the analyzed antigens. All immunoblots are given in the Supplementary Data (section Supplementary Materials) and noted from Figures S1–S7.

While the assay by Immunofluor CHAGAS gave five false negatives in Group 1 and 30 false positives, Chagatest ELISA Recombinante v. 4.0 gave one false negative and 18 false positives (Table 2).

We calculated the sensitivity and the specificity as a measure of the diagnostic performance of the test. The results are given before and after the calculation of the confidence interval, according to the Wilson score interval with continuity correction. 

For the Chagas Western Blot IgG assay, we calculated a sensitivity of 100% (95% CI: 95.3–100) and a specificity of 100% (95% CI: 97–100) (Table 3).

In contrast, conventional serology using synthetic peptides (Chagatest ELISA Recombinante v4.0) or whole *T. cruzi* inactivates (Immunofluor CHAGAS) showed lower sensibilities, respectively, of 99% (95% CI: 93.8–100) and 95% (95% CI: 88.2–98.1) and lower specificity indexes of 91.3% (95% CI: 85.9–91.8) and 83.1% (95% CI: 76.6–88.2), respectively, mainly due to cross-reactions with sera from Leishmaniasis. 

### 2.3. The Chagas Blot on Serum with Discordant Serological Results

**Figure 1:**
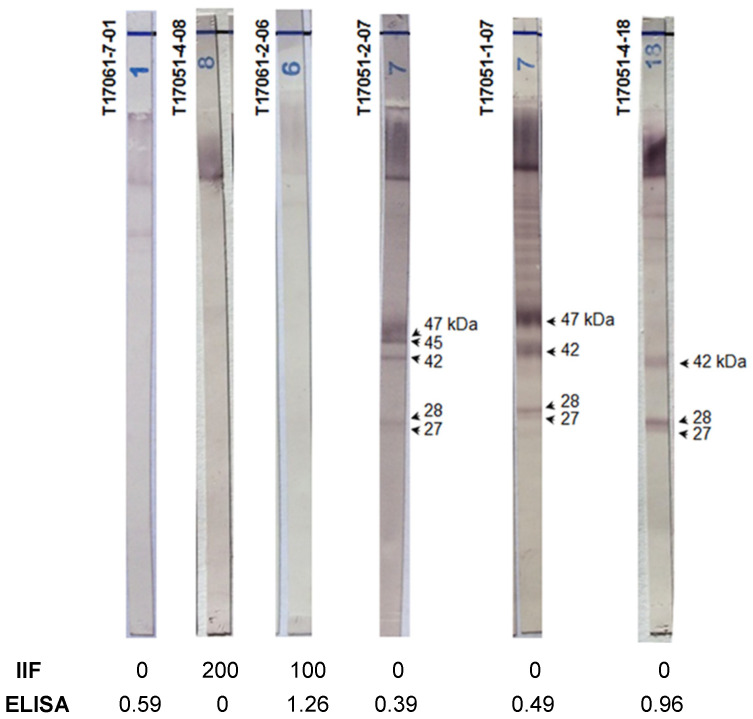
The Chagas blot on serum clarified the discordant serological results. T17061-7-01: serum from a patient with leishmaniosis, T17051-04-08: serum from a 53-year-old French woman without anti-*T. cruzi* antibodies, T17061-2-06: serum from a 48-year-old French man with visceral leishmaniosis, T1-7051-2-07: serum from a 6-month-old baby born from a mother with Chagas disease, T17051-01-07: serum from a 35-year-old Brazilian man with Chagas disease and T17051-4-18: serum from a 50-year-old woman with Chagas disease.

Two different diagnostic tests (IIF and ELISA) gave discordant results for 54 of the 278 (19.4%) sera in our study (Groups 1 and 2 combined). We performed the Chagas blots on those sera, and the results enabled the correct of their classification. Figure 1 shows, for example, six immunoblots performed with three sera of Groups 1 and 2. Concerning Group 2, the serological results for the first serum were negative using IIF and weakly positive using ELISA. In contrast, the second serum was positive using IIF and negative using ELISA. The last serum, from a patient with visceral leishmaniasis, was positive using IIF and positive using ELISA. However, the immunoblot patterns (T17061-7-01, T17051-4-08 and T17061-2-06) of these sera were clearly negative. Concerning Group 1, the serological results were negative using IIF and weakly positive using ELISA. Again, and in contrast, the immunoblots showed good reactions with at least two protein bands (T17051-2-07, T17051-1-07 and T17051-4-18). 

Thus, in the present study, the Chagas blot was able to confirm the presence or absence of *T. cruzi* antibodies, with a probability of 100%, for patients presenting discrepant (and, thus, inconclusive) results from other serological methodologies.

### 2.4. Chagas Blot Performed on Sera from Newborns

**Figure 2:**
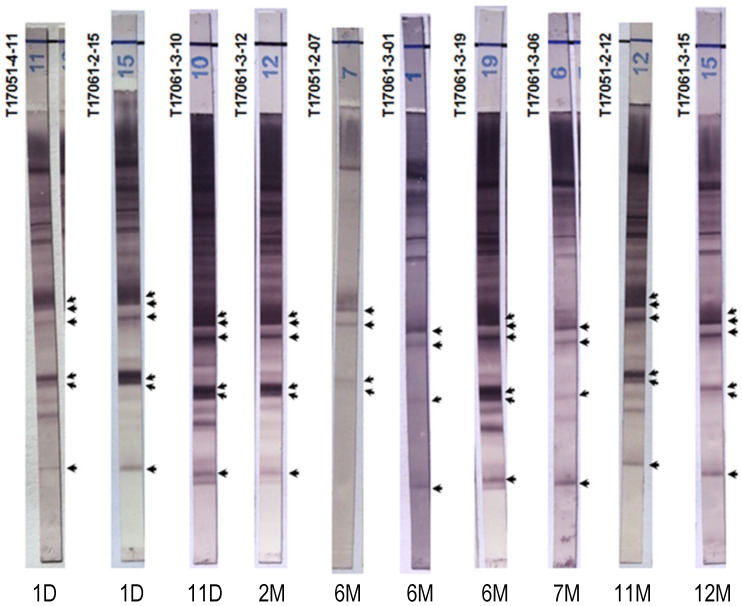
Chagas blot on sera from newborns. The ages of the patients are marked below each immunoblot, day (D) and month (M). T17051-4-11 and T17061-2-15: sera from 1-day-old newborns. T17051-2-07: serum from a 6-month-old baby. These three babies were born in France to three Bolivian mothers with Chagas disease. T17061-2-12: sera from an 11-month-old baby. T17061-3-10, 12, 01, 19, 06, 15 and T17051-2-12: sera, respectively, from newborns/babies aged 11 days, 2 months, 6 months, 6 months, 7 months, 11 months and 1 year. Babies born in Argentina to mothers with Chagas disease were treated at the Ricardo Gutiérrez Children’s Hospital. Arrows indicate the protein bands specifying the presence of anti-*T. cruzi* antibodies.

Specifically, the sera of children aged less than one year (from Argentina (*n* = 7) and France (*n* = 3)) showed 100% positivity using the Chagas blot (Figure 2). However, the patterns of T17051-2-07, T17061-3-01 and T17061-3-06 were less complex than the others and particularly with immunoblot T17061-3-19 realized with serum from a baby at the same age. As expected, the immunoblot patterns of newborns were similar to those of their mothers, except for the first three newborns, for whom the patterns showed a lower intensity. As shown above, the serological results of the ELISA and IIF tests for T17051-2-07 were discordant. In this case, the mother’s anti-*T. cruzi* antibodies were less abundant or eliminated, but the immunoblot nonetheless gave a positive result. This immunoblot revealed only the presence of anti-*T. cruzi* antibodies and not the signature of an active infection. Despite this, the results obtained showed the very high sensitivity of this test, since, with it, we were able to reveal traces of antibodies.

### 2.5. Chagas Blot Performed on Sera with Other Parasitic Diseases

**Figure 3:**
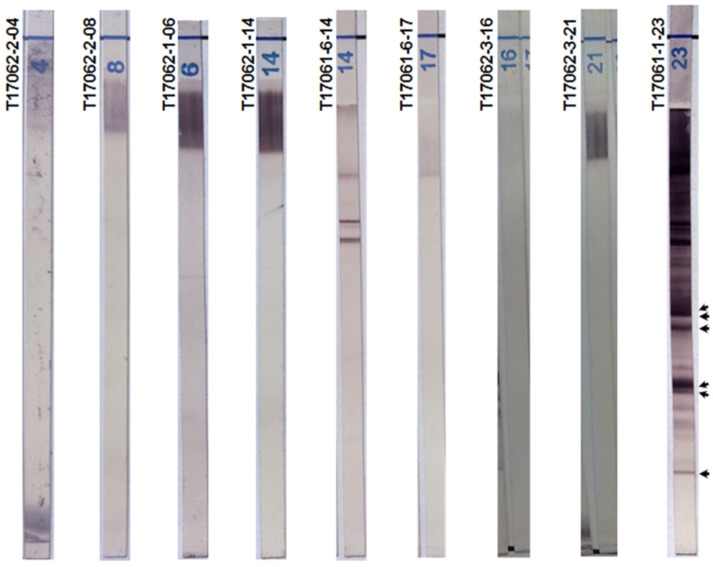
Chagas blot on other parasitic diseases. T17062-2-04 and 08: sera, respectively, from a 40-year-old French woman and a 25-year-old French woman, both with toxoplasmosis. T17062-1-06 and 14: sera, respectively, from a 77-year-old man and a 20-year-old woman, both with malaria. T17061-6-14 and 17: sera with leishmaniasis. T17062-3-21 and 23: sera, respectively, from a 45-year-old woman and 45-year-old man, both with visceral amebiasis. T17061-1-23: serum from a 48-year-old Bolivian woman with Chagas disease (positive reference). Arrows indicate the protein bands specifying the presence of anti-*T. cruzi* antibodies.

We performed the Chagas blot for many patients with leishmaniosis (*n* = 44), malaria (*n* = 22), toxoplasmosis (*n* = 27) and amebiasis (*n* = 6). None of these sera gave a positive result with the immunoblot (Figure 3). Furthermore, there were no protein bands detectable in the concerned immunoblots with respect to the positive reference. This result suggests a remarkable specificity for the Chagas blot.

## 3. Discussion

According to the WHO 2002 recommendations [3], the diagnosis of chronic cases should involve two different serological methods with different antigens (immunoassays and indirect immunofluorescence (IIF) assays). The sensitivity of these approaches has been established, but false-positive results may occur due to cross-reactions with other parasitic diseases, especially leishmaniasis [4]. Until now, the indirect diagnosis of Chagas disease has lacked a reliable gold standard. Ideally, this latter is a serological test offering a definite diagnosis with a simple and achievable use in routine diagnosis.

Serological techniques are based on whole parasite antigens and purified extracts (conventional tests) and on recombinant antigens and synthetic peptides (non-conventional tests) [5]. 

As stated in WHO’s second report published in 2002 [3], the current recommendation for the indirect diagnosis of Chagas disease is the use of two conventional, methodologically different serological tests simultaneously to detect *T. cruzi* antibodies. If only one method is used, it should involve different antigens (native versus recombinant antigens) [3]. If the results obtained by the two initial tests are not concordant, a third test should be performed. Three conventional tests are widely used: indirect hemagglutination (IHA), indirect immunofluorescence (IIF) and enzyme immunoassay (EIA). There is no gold standard for the diagnostic of Chagas disease, although a radioimmunoprecipitation assay, an in-house technique from the University of Iowa [6], has been used as a confirmatory test in several ongoing and published studies of *T. cruzi* [7,8]. However, radioimmunoprecipitation is a difficult technique intended only for research, not used routinely for diagnostic purposes.

Previous studies have reported a high sensitivity and specificity for several serological tests intended for Chagas disease diagnosis [8,9,10,11].

In 2010, a WHO-organized multicenter study [12] compared 24 commercial assays for the diagnosis of Chagas Disease on a panel of 437 sera. That study showed that ELISA outperformed IHA, offering a better sensitivity (98.6% (94.0–100%) versus 96.3% (88.09–100%)) and specificity (98.9% (97.6–100) versus 84.0 (59.9–99.6%)). EIA also had a lower, but not insignificant, percentage of indeterminate results (0.65 % (0–1.83%)) in comparison to IHA (1.28% (0.23–3.20%)). In France, a non-endemic area, a study in 2011 by the French Blood bank [13] showed that, among the 163,740 donations that had been tested for Chagas disease (3.5% of the total donations), five were positive and close to 1400 indeterminate (0.85%). In Peru, a recent study [14] showed that, of 7723 donations evaluated for Chagas disease in 2014 to 2015, ten were positive (overall prevalence 0.14%) and 98 indeterminate (overall prevalence 1.27%). In both of these examples, the number of indeterminate results was superior to that of the positive results. Consequently, the relatively high percentage of indeterminate results is a serious problem in the context of blood donations and blood transfusions, where it results in a large volume of discarded blood and an increase in costs. The new chemiluminescence and electroluminescence assays, as suggested by several studies [15,16,17,18], are probably more specific and sensitive than IIF ant ELISA, but there is a real need to reduce the rate of indeterminate results, either through improvement of the current diagnostic tests or the development of a reliable confirmatory test. 

Here, we evaluated the Chagas Western Blot IgG assay (Chagas blot), an immunoblot based on the detection of anti-*T. cruzi* antibodies. The sensitivity and specificity of the Chagas blot were both 100% in our collection of 278 sera.

In our study, 48 sera from Group 1 (presence of anti-*T. cruzi* antibodies) showed discordant results using two conventional assays but were correctly classified using the Chagas blot.

Among those discordant results was the serum from a 6-month-old baby born in France from a Bolivian mother with Chagas disease. The IIF assay was negative, the ELISA weakly positive and the Chagas blot was undoubtedly positive (presence of five bands). Interestingly, the positivity of the test was not related to congenital Chagas disease but, rather, to the persistence of maternal antibodies in the newborn.

In-line with previously reported assays [19,20], we found that more than half of the indeterminate results of the control group by the IIF/EIA methods originated from patients with leishmaniosis. Leishmaniosis and other parasitic diseases such as amebiasis, malaria and toxoplasmosis did not provoke the appearance of bands with the Chagas blot, demonstrating a very high specificity of the immunoblot. 

The results we presented here suggest that the Chagas blot is particularly sensitive and specific. Nonetheless, they must be confirmed in larger tests involving many sera from different regions of South America before this immunoblot can be considered as a universal confirmatory test. Indeed, the Chagas blot is made with crude extracts from discrete typing unit (DTU) TcVI *T. cruzi* (CL Brener strain). The sera in our study were taken mainly from patients in Southern-Central South America (Bolivia, Argentina, Paraguay and Chile), with the exception of one patient from Northern South America (Colombia), and were not genotyped. 

Some twenty years ago, an immunoblot assay based on a *T. cruzi* protein antigen fraction from epimastigotes bound to a nitrocellulose membrane was proposed as a supplementary serological test for Chagas disease [21] More recently, another immunoblot was proposed, this one based on a trypomastigote excreted–secreted antigen (TESA) fraction produced in cultures of *T. cruzi*-infected (strain Y) mammalian cells [22,23]. The presence of bands in the 120–200-kDa molecular mass region indicated a positive result and the absence of such bands a negative result. We evaluated the TESA blot six years ago and found sensitivity and specificity rates of approximately 100% (unpublished data), in alignment with the results published by the WHO in 2010 [12] or others [24,25,26,27,28,29]. Neither of these two tests have been developed commercially.

There are, however, two other commercial immunoblots for the diagnosis of Chagas disease that are not available in Europe. The HBK 740 Immunoblot Linhas anti-*T. cruzi*, developed by Empresa Brasileira de Biotecnologia (EMBRABIO [11], and the Abbott ESA Chagas (Abbott) [30,31]. These assays employ recombinant antigens and show high sensitivity and specificity. The time needed to perform the HBK 740 is rather long (18 h). The Abbott ESA Chagas has been approved by the American FDA for the confirmation of blood donors who are repeatedly reactive on Chagas screening tests. 

## 4. Materials and Methods

Study design. We performed a retrospective case–control study including 278 patients that came from the Parasitology–Mycology laboratory of the Pitié-Salpêtrière Hospital (Paris, France) and the Laboratory of Parasitology and Chagas Disease of the Ricardo Gutiérrez Children’s Hospital (Buenos Aires, Argentina). The study was conducted according to the Declaration of Helsinki. Case definitions were based on a combination of clinical presentation, anamnestic data and biological results.

Antibody detection. All samples were tested in parallel with an enzyme immunoassay based on the use of recombinant *T. cruzi* proteins (Chagatest ELISA Recombinante v.4.0; Wiener Lab, Rosario, Argentina), an indirect immunofluorescence assay that use inactivated parasites (Inmmunofluor Chagas; Biocientífica S. A., Buenos Aires Argentina) and the Chagas Western Blot IgG Assay^®^ (LDBio Diagnostics, Lyon, France). Assay determinations with the commercial kits were performed according to the manufacturer’s instructions.

## 5. Conclusions

The new Chagas Western Blot IgG assay is a powerful, easy-to-use test for detecting specific anti-*Trypanosoma cruzi* antibodies for the diagnosis of chronic forms of Chagas disease. Moreover, it is able to clarify results remaining equivocal after the use of conventional assays and, thus, has great potential as a confirmational assay. Further studies in many diagnostic centers and on many patients are, however, needed to confirm the results reported here.

## Figures and Tables

**Table 1:** Sample description.

		Anti-*Trypanosoma cruzi* Antibodies Detected	Anti-*Trypanosoma cruzi* Antibodies Not Detected
Group		Group 1	Group 2
Sample	Serum (N)	100	178
Gender	Male (%)	24 (24%)	78 (44%)
	Female (%)	73 (73%)	55 (31%)
	Not Available	3 (03%)	45 (25%)
Age	Median	34 years	37 years
	Average	32 years	38 years
	Limits	(1 day–78 years)	(10 months–77 years)
Origin		Endemic areas	Non endemic areas

**Table 2:** Diagnostic results obtained with the Chagas blot in comparison with two other commercial assays.

Test	Group 1 (*n* = 100)		Group 2 (*n* = 178)	
	True Positive	False Negative	True Negative	False Positive
Chagas Western Blot IgG assay	100	0	178	0
Immunofluor CHAGAS	95	5	148	30
Chagatest ELISA Recombinante v.4,0	99	1	160	18

**Table 3:** Sensitivity and specificity of the Chagas blot in comparison with two other commercial assays.

Test	Sensitivity	Specificity
	*n* = 100	*n* = 178
	(95% CI)	(95% CI)
Chagas Western Blot IgG assay	100	100
	(95.3–100)	(97.4–100.0)
Immunofluor CHAGAS	95.0	83.1
	(88.2–98.1)	(76.6–88.2)
Chagatest ELISA Recombinante v.4.0	99.0	91.3
	(93.8–100.0)	(85.9–91.8)

## Data Availability

Not applicable.

## Supplementary Materials

The following are available online at https://www.mdpi.com/article/10.3390/pathogens10111455/s1: Figure S1: Immunoblot performed with Chagas blot, kit T1-7051-1. Figure S2: Immunoblot performed with Chagas blot, kit T1-7051-2. Figure S3: Immunoblot performed with Chagas blot, kit T1-7051-3. Figure S4: Immunoblot performed with Chagas blot, kit T1-7051-4. Figure S5: Immunoblot performed with Chagas blot, kit T1-7061-1. Figure S6: Immunoblot performed with Chagas blot, kit T1-7061-2. Figure S7: Immunoblot performed with Chagas blot, kit T1-7061-3. Figure S8: Immunoblot performed with Chagas blot, kit T1-7061-5. Figure S9: Immunoblot performed with Chagas blot, kit T1-7061-6. Figure S10: Immunoblot performed with Chagas blot, kit T1-7061-7. Figure S11: Immunoblot performed with Chagas blot, kit T1-7062-1. Figure S12: Immunoblot performed with Chagas blot, kit T1-7062-3.

## Abbreviations

The following abbreviations are used in this manuscript:

IHA	Hemagglutination
IIF	Indirect Immunofluorescence
EIA	Enzyme Immunoassay
ELISA	Enzyme-Linked Immunosorbent Assay
WHO	World Health Organization,
NBT-BCIP	Nitrotetrazolium blue chloride. 5-bromo-4-chloro-3-indolyl-phosphate-p-toluidine salt
DTU	Discrete Typing Unit

## Appendix A

The Chagas Western Blot IgG assay (Chagas blot) was developed and standardized by LDBio Diagnostics. The present study was performed between June 2017 and October 2017. Before using the Chagas blot, and with the aim of increasing the homogeneity of the diagnostic results, all the samples were tested in parallel, with (i) Chagatest ELISA Recombinante v.4.0 (Wiener Lab, Rosario, Argentina), an enzyme immunoassay based on the use of recombinant *T. cruzi* proteins (Ag1, Ag2, Ag13, Ag30, Ag36 and SAPA) as antigens, and with (ii) Inmmunofluor Chagas (Biocientífica S. A., Buenos Aires, Argentina), an IIF assay that employs whole inactivated parasites. Assay determinations with the commercial kits were performed according to the Chagas blot manufacturer’s instructions.

The Chagas blot was performed using native trypomastigote and amastigote antigens from the CL Brener *T. cruzi* strain described above. Proteins were extracted, separated by SDS PAGE (polyacrylamide gel electrophoresis) and transferred to a nitrocellulose membrane. The native antigens coupled to this latter were used to detect the presence of anti-*T. cruzi* antibodies. Anti-immunoglobulin antibodies coupled to a conjugate were added to detect the captured *T. cruzi* antibodies. A substrate was added thereafter (NBT-BCIP) to react with the conjugate and reveal the presence of parasitic protein bands. All the tests were performed by a single person but interpreted by at least two investigators. The immunoblots were performed on the EUROBlotmaster (EUROIMMUN), according to the manufacturer’s protocols.

Criteria for sample positivity

The positivity criteria of the immunoblot used in this study were those given by the manufacturer; they are given briefly below. For the Chagas blot, a positive sample can have many bands between 2 and 200 kilo Daltons (kDa). However, the reading area is at the bottom of the strip, between 10 and 50 kDa. Five bands are frequently present: P15–16, P21–22, P27–28, P42 and P45–47, corresponding, respectively, to molecular weights of 15, 21, 27, 42 and 45 kDa. The appearance of bands varies. Notably, bands P15–16, P21–22 and P27–28 may appear as a single broad band, a doublet of two thin bands or one of the two component bands of the doublet.

For the study presented here, we considered that the presence of a minimum of two of these five frequently present bands indicated the presence of anti-*T. cruzi* antibodies in the sample.

Sensitivity and specificity

The sensitivity (Se) and specificity (Sp) were, respectively, defined as the proportion of samples considered positive or negative for the presence of anti-*T. cruzi* antibodies correctly identified as positive or negative by the Chagas blot. We calculated the Se and Sp as follows: Se = true positives/(true positives + false negatives) and Sp = true negatives/(true negatives + false negatives).
