# Supplementary material for: Evaluation of the Chagas Western Blot IgG Assay for the Diagnosis of Chagas Disease
Source: Pathogens. 2021 Nov 10;10(11):1455. doi: 10.3390/pathogens10111455 (PMC8624453; doi:10.3390/pathogens10111455)
Supplement: Supplementary file 1 [file pathogens-10-01455-s001.zip › pathogens-1402736-supplementary.pdf]

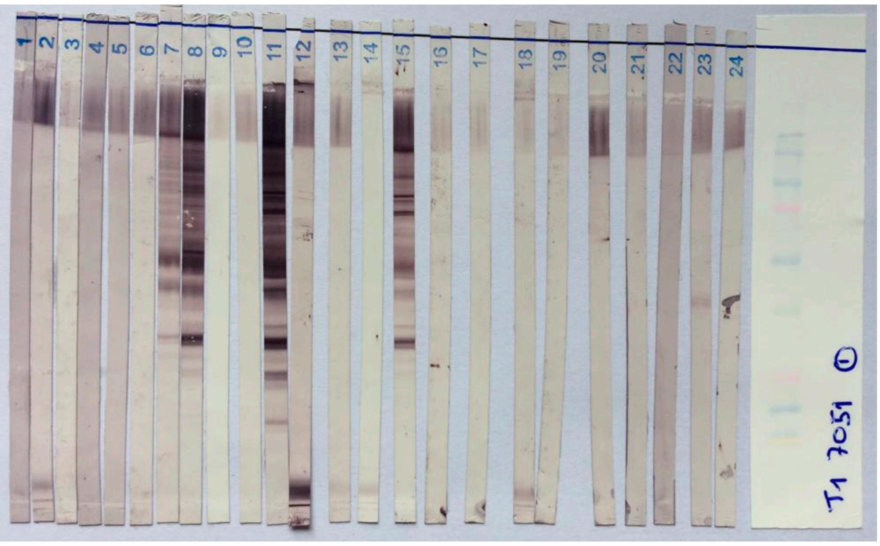

Figure S1: Immunoblot performed with Chagas blot, kit T1-7051-1.

Strips 7, 8, 11 and 15 were done on sera from patients with anti-*T cruzi* antibodies. Strip 1 to 6, 9, 10, 12 to 14, and 16 to 24 were done on sera from control patients.

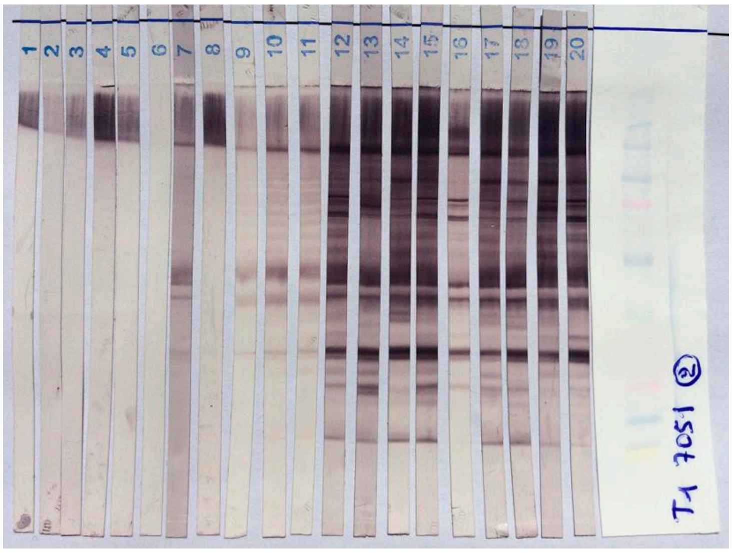

Figure S2: Immunoblot performed with Chagas blot, kit T1-7051-2.

Strips 1 to 6 and 8 were done on sera from control patients. Strip 7 was done on serum with anti-*T cruzi* antibodies. Strips 9 to 11 were done with increasing volumes of serum (10, 20 and 50 µL) from a same patient with anti-*T cruzi* antibodies. Strips 12 to 20 were done with sera from patients with Chagas disease and at the Ricardo Gutierrez Hospital (BA, Argentina).

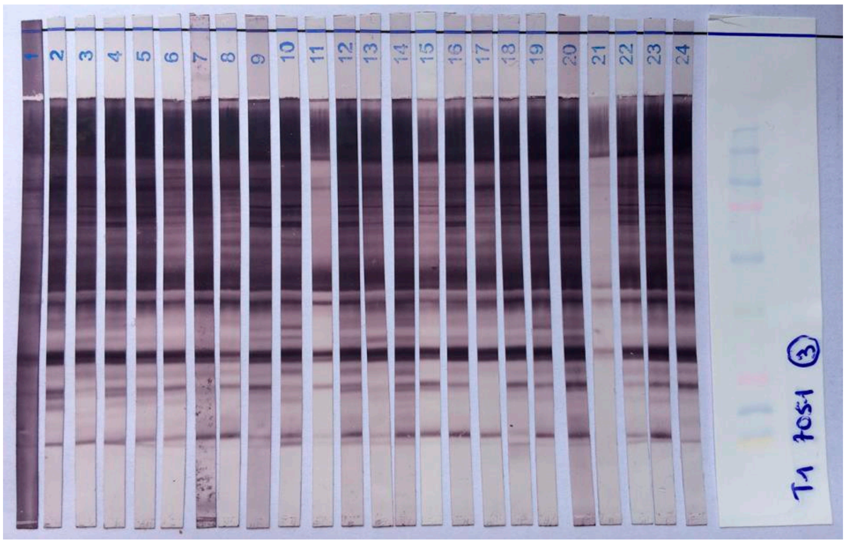

Figure S3: Immunoblot performed with Chagas blot, kit T1-7051-3.

Strips 1 to 24 were done on sera (20 μL) from patients with Chagas disease and at the Ricardo Gutierrez Hospital (BA, Argentina).

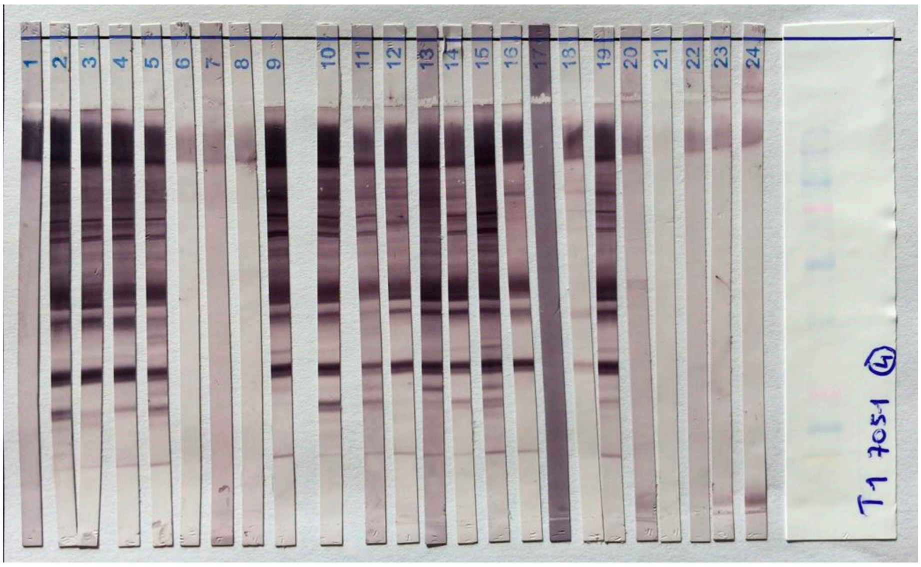

Figure S4: Immunoblot performed with Chagas blot, kit T1-7051-4.

Strips 2 to 5, 9 to 16, 18 and 19 were done on sera (20 μL) from patients with Chagas disease and at the Pitié Salpêtrière hospital. Strips 1, 6 to 8, 17, and 20 to 24 were done on control sera (20 μL) from patients free of Chagas disease.

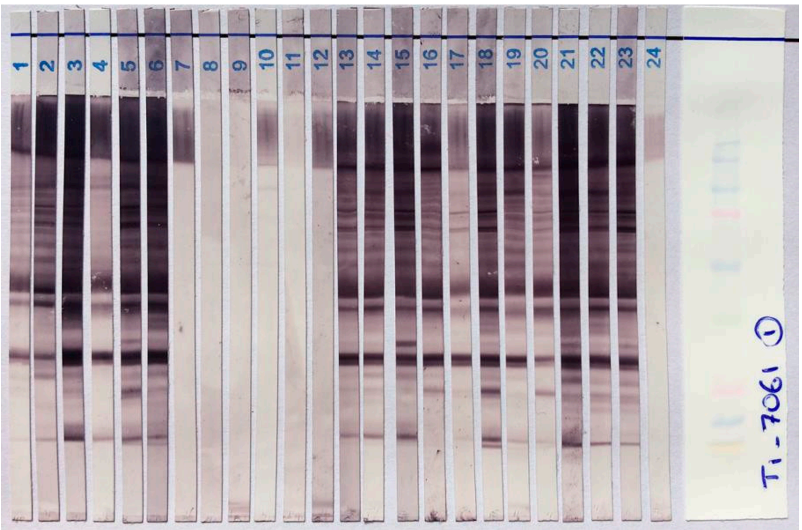

Figure S5: Immunoblot performed with Chagas blot, kit T1-7061-1.

Strips 1 to 6, 13 and 24 were done on sera (20 µL) from patients with Chagas disease and at the Pitié Salpêtrière hospital. Strips 7 to 12 were done on control sera (20 µL) from patients free of Chagas disease.

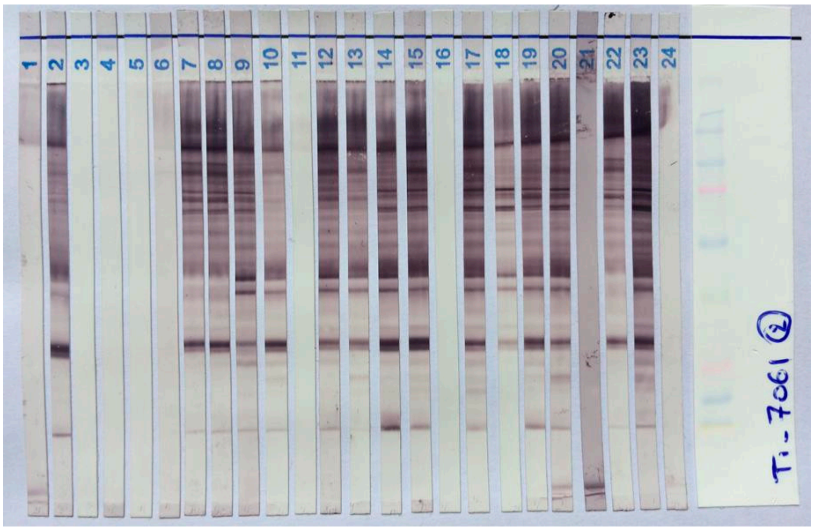

Figure S6: Immunoblot performed with Chagas blot, kit T1-7061-2.

Strips 1, 3 to 6, 11, 16 and 21 were done on sera (20 µL) from control patients free of CD and at the Pitié Salpêtrière Hospital (Paris, France). Strips 2, 9, 10, 12 to 15, and 17 to 20 were done on sera (20 µL) from test patients with CD and at the Pitié Salpêtrière Hospital (Paris, France). Strips 7, 8 and 22 were done on external laboratory quality-control sera. Strip 23: positive control: serum from a 48-year-old Bolivian woman with CD. Strip 23: negative control.

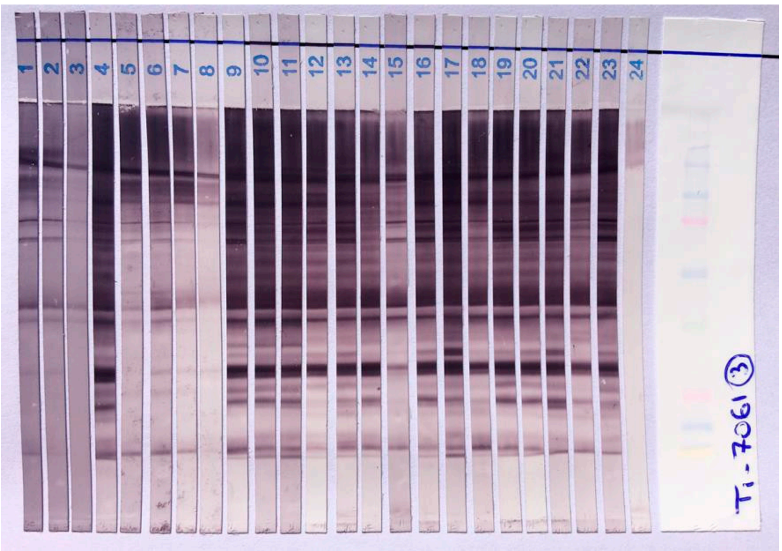

Figure S7: Immunoblot performed with Chagas blot, kit T1-7061-3.

Strips 1 to 22 were done on sera from patients with Chagas disease and at the Ricardo Gutierrez Hospital (BA, Argentina). The first three strips were done on sera from a same 6-month-old girl, born to a mother with Chagas disease (Strip 4), before benznidazole treatment (strip 1), 1 and 7 month after the end treatment (strip 2 and 3).

Strips 5 to 8 were done on sera from a same 6-month-old girl, born to a mother with Chagas disease (Strip 9), born to a mother with Chagas disease (Strip 4), before benznidazole treatment (strip 5), 1 and 2 and 10 month after the end treatment (strip 6, 7 and 8).

Strip 10 was done on serum from a 1-day-old infant born to a mother with Chagas disease (Strip 11). Strip 12 was done on serum from a 2-month-old infant born to a mother with Chagas disease (Strip 13). Strip 14 was done on serum from a 15-year-old girl with Chagas disease. Strip 15 was done on serum from a 1-year-old boy born to a mother with Chagas disease (Strip 16). Strip 19 was done on serum from a 6-month-old infant born to a mother with Chagas disease (Strip 20). Strips 17, 18, 21 and 22 were done on sera from patients with Chagas disease. Strip 23: positive control: serum from a 48-year-old Bolivian woman with CD. Strip 23: negative control.

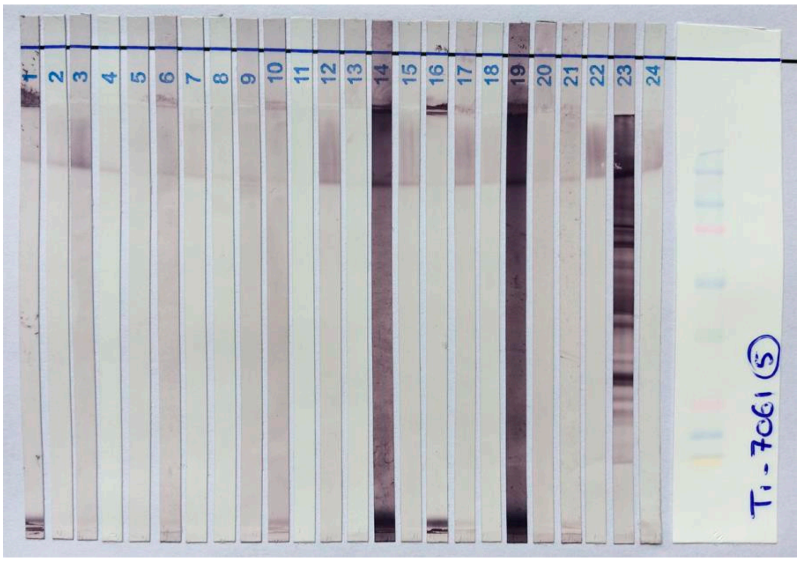

Figure S8: Immunoblot performed with Chagas blot, kit T1-7061-5.

Strips 1 to 22 were done on sera (20 µL) from patients with no anti-*T. cruzi* antibodies. Strip 23: positive control: serum from a 48-year-old Bolivian woman with CD. Strip 23: negative control.

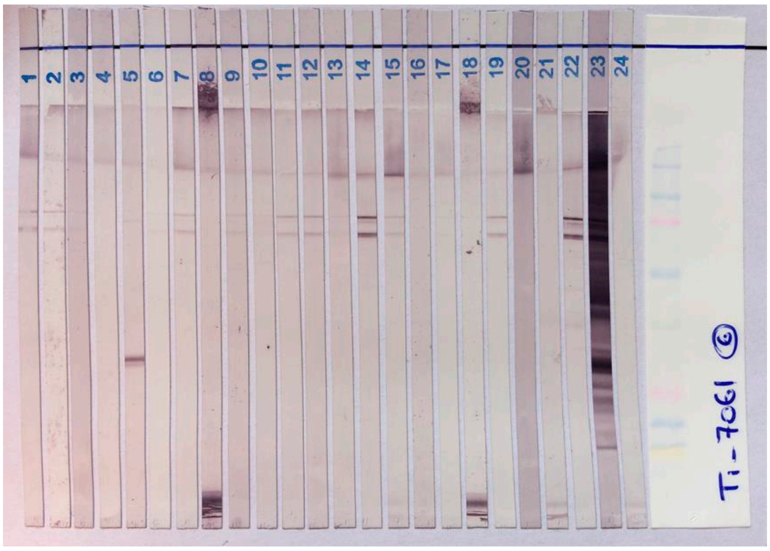

Figure S9: Immunoblot performed with Chagas blot, kit T1-7061-6.

Strips 1 to 22 were done on sera (20 µL) from leishmaniosis patients. Strip 23: positive control: serum from a 48-year-old Bolivian woman with CD. Strip 23: negative control.

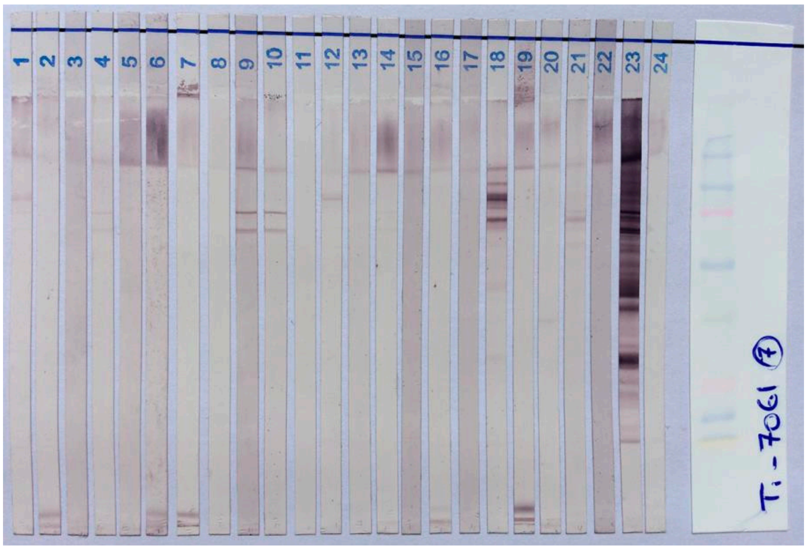

Figure S10: Immunoblot performed with Chagas blot, kit T1-7061-7.

Strips 1 to 22 were done on sera (20 µL) from leishmaniosis patients. Strip 23: positive control: serum from a 48-year-old Bolivian woman with CD. Strip 23: negative control.

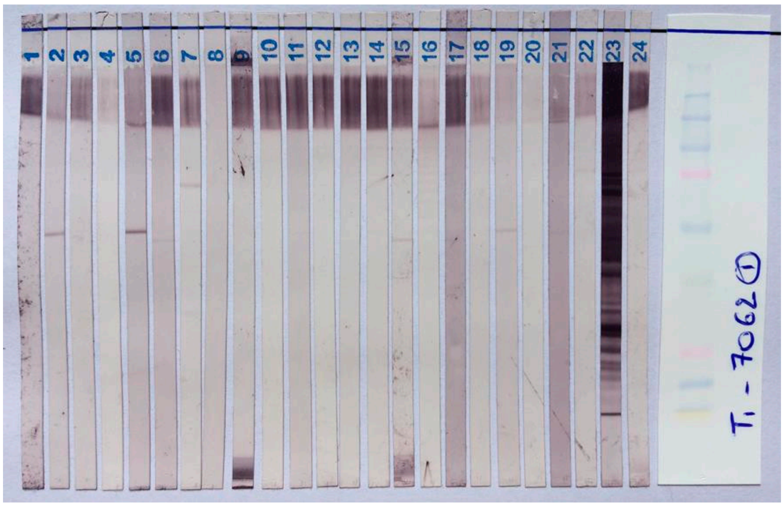

Figure S11: Immunoblot performed with Chagas blot, kit T1-7062-1.

Strips 1 to 22 were done on sera (20 µL) from malaria patients. Strip 23: positive control: serum from a 48-year-old Bolivian woman with CD. Strip 23: negative control.

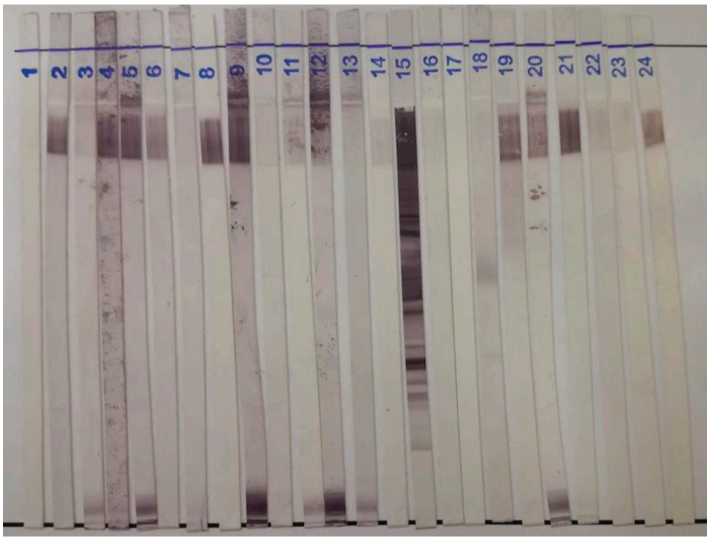

Figure S12: Immunoblot performed with Chagas blot, kit T1-7062-3.

Strips 1 to 14 were done on sera from patients with toxoplasmosis. Strip 15: positive control: serum from a 48-year-old Bolivian woman with CD. Strip 18 was done on a patient with leishmaniosis. Strips 16, 17 and 19 to 23 were done on sera from patients with amebiasis.
